# Manipulation of *Salmonella* Typhi Gene Expression Impacts Innate Cell Responses in the Human Intestinal Mucosa

**DOI:** 10.3389/fimmu.2018.02543

**Published:** 2018-11-01

**Authors:** Rosângela Salerno-Gonçalves, James E. Galen, Myron M. Levine, Alessio Fasano, Marcelo B. Sztein

**Affiliations:** ^1^Center for Vaccine Development and Global Health, University of Maryland School of Medicine, Baltimore, MD, United States; ^2^Mucosal Immunology and Biology Research Center, Massachusetts General Hospital for Children, Boston, MA, United States

**Keywords:** mucosal immunity, innate immunity, Salmonella, typhoid vaccine, gut, human

## Abstract

Although immunity induced by typhoid fever is moderated and short-lived, typhoid vaccination with the attenuated Ty21a oral vaccine generates long-lasting protection rates reaching up to 92%. Thus, there are important differences on how wild-type *Salmonella* and typhoid vaccine strains stimulate host immunity. We hypothesize that vaccine strains with different mutations might affect gut inflammation and intestinal permeability by different mechanisms. To test this hypothesis, we used an *in vitro* organotypic model of the human intestinal mucosa composed of human intestinal epithelial cells, lymphocytes/monocytes, endothelial cells, and fibroblasts. We also used six *Salmonella enterica* serovar Typhi (*S*. Typhi) strains: the licensed Ty21a oral vaccine, four typhoid vaccine candidates (i.e., CVD 908, CVD 909, CVD 910, and CVD 915) and the wild-type Ty2 strain. We found that genetically engineered *S*. Typhi vaccine strains elicit differential host changes not only in the intestinal permeability and secretion of inflammatory cytokines, but also in the phenotype and activation pathways of innate cells. These changes were distinct from those elicited by the parent wild-type *S*. Typhi and depended on the genetic manipulation. In sum, these results emphasize the importance of carefully selecting specific manipulations of the *Salmonella* genome in the development of typhoid vaccines.

## Introduction

It is widely accepted that immunity induced by typhoid fever is moderate and short-lived, and recurrences after re-exposure to the infection are typical (1, 2). Interestingly, protection rates can reach up to 92% for the Ty21a oral vaccine depending on the number of vaccine doses and formulation and last for up to 7 years (3–5). Thus, it is reasonable to hypothesize that there are significant differences on how wild-type *Salmonella* and attenuated typhoid vaccine strains stimulate the host immune responses. Gut innate cells are known to play a vital role in shaping adaptive immune responses. Migration of activated B and T cells is governed by cytokines and chemokines produced by innate cells, such as macrophages, endothelial and epithelial cells (6). Indeed, analyses of the genes implicated in inflammatory bowel disease have highlighted several pathways that are crucial for intestinal homeostasis, including barrier function, epithelial repair, microbial defense and innate immune regulation (7). Yet, the mechanism by which innate immune responses in the gut shape adaptive immunity to vaccines remains to be further explored. We hypothesize that wild-type *S*. Typhi and isogenic vaccine strains with different mutations might influence the gut inflammation and epithelial cell barrier function by different means.

To address this hypothesis we used a multicellular organotypic model of the human intestinal mucosa composed of human intestinal epithelial cells, lymphocytes/monocytes, endothelial cells, and fibroblasts (8–10). We also used six closely related *Salmonella enterica* serovar Typhi (*S*. Typhi) strains: the wild-type Ty2, the licensed Ty21a oral vaccine, and four typhoid vaccine candidate strains (i.e., CVD 908, CVD 909, CVD 910, and CVD 915) that were developed at the Center for Vaccine Development (CVD), University of Maryland (Table 1). The attenuation of these typhoid vaccine candidates is based on deletions of genes, such as *aroC, aroD, htrA*, and *guaBA*. Two out of the four vaccine candidates used in this study, designated CVD 908 (11), and CVD 909 (12), have been tested in volunteers (13) representing an exceptional tool to investigate gut innate immune responses. While both CVD 908 and CVD 909 were highly immunogenic and non-reactogenic, CVD 908 induced a clinically silent, self-limited vaccine bacteremia. All four vaccine candidate strains are derived from the parent wild-type *S*. Typhi strain Ty2. The licensed oral Ty21a vaccine was used as the “gold standard.” Ty21a is also derived from the *S*. Typhi strain Ty2 and has shown good immunogenicity and an excellent record of safety and tolerability in humans (5, 14, 15). Using a 3-D model of the human intestinal mucosa we found that genetic engineering of *S*. Typhi vaccine strains elicits host changes not only in the intestinal permeability and secretion of inflammatory cytokines but also in the phenotype and activation pathways of innate cells. These changes are distinct from those elicited by the parent wild-type Ty2 *S*. Typhi strain and are dependent on the genetic manipulation. Thus, these results emphasize the impact of carefully selecting specific manipulations of the *Salmonella* genome for the development of attenuated typhoid vaccines.

**Table 1:** Description of *S*. Typhi strains used in the manuscript.

**Serovar Typhi strain**	**Parent strain**	**Relevant phenotype**	**Metabolic pathway affected**	**Phenotype *in vitro***	**Field/Clinical trial performance**
Ty2	Wild-type	–	–	Rapid multiplication	No or short lived protection
Ty21a	Ty2	Chemical mutagenesis	Several critical metabolic pathways, such as galactose metabolism, synthesis of isoleucine, valine, and Vi-capsular PS (see text for details)	Limited multiplication	Up to 7 years of protection
CVD 908	Ty2	ΔaroC ΔaroD	Biosynthesis of aromatic aa	Limited multiplication	Immunogenic but with clinically silent, self-limited vaccine bacteremia
CVD 909	Ty2 CVD 908-htrA	ΔaroC ΔaroD ΔhtrA Ptac-tviA	Biosynthesis of aromatic aa and heat-shock protein. Constitutively express Vi	Limited multiplication	Immunogenic
CVD 910	Ty2 CVD 908-htrA	ΔguaBA ΔhtrA	Biosynthesis of heat-shock protein Biosynthesis of guanine nucleotides	Limited multiplication	No clinical trials
CVD 915	Ty2	ΔguaBA	Biosynthesis of guanine nucleotides	Limited multiplication	No clinical trials

## Materials and methods

### Ethics statement

All blood specimens were collected from volunteers who participated in the University of Maryland Institutional Review Board approved protocol (number HP-00040025) that authorized the collection of blood specimens from healthy volunteers for the studies included in this manuscript. This protocol has been conducted following the ethical standards laid down in the 1964 Declaration of Helsinki and its later amendments. Volunteers were explained the purpose of this study and gave informed, signed consent before the blood draw. Peripheral blood mononuclear cells (PBMC) were isolated from the blood by density gradient centrifugation and cryopreserved in liquid N_2_ following standard techniques (8, 16).

### 3-D model cells and culture media

The 3-D model system comprised of the human intestinal epithelial cell line HCT-8 cells (CCL-244, ATCC, Manassas, VA) and primary human lymphocytes/monocytes, endothelial cells (HUVEC cells, CRL-1459, ATCC), and fibroblasts (CCD-18Co cells, CRL-1459, ATCC) was cultured under microgravity conditions. Cell cultivation and the set-up of the 3-D model were performed as previously described (8–10, 17). Briefly, fibroblasts and endothelial cells were embedded in an enriched collagen-I matrix (Invitrogen, Carlsbad, CA) and added together with epithelial cells to the rotating wall vessels (RWV) (Synthecon, Houston, TX) that provided microgravity conditions to the cultures. Peripheral blood mononuclear cells (PBMC) enriched in lymphocytes/monocytes isolated from healthy adult volunteers (2 × 10^7^/vessel) were added to the 3-D model culture at days 4 and 9 (± 1 days) (8–10, 17). PBMC were isolated by Ficoll density gradient centrifugation, and added to the cultures without stimulation. Thus, these PBMC have the same frequencies of cell subsets as those observed in circulation in healthy adults, e.g., 70–90% lymphocytes, 10–20% monocytes and 1–2% dendritic cells (18). The experiments were performed with 15–18 days-old 3-D models after the cells within the 3-D model were fully differentiated.

### Bacteria description, media and growth conditions

Fully virulent *S*. Typhi wild-type strain Ty2 was used in these experiments; all live attenuated strains used were derived from the Ty2 parent strain (see Table 1). The licensed typhoid vaccine Ty21a was derived from Ty2 by chemical mutagenesis and carries attenuating mutations in multiple genes, including *galE, galT, alK, galP, rpoS, ilvD, rcsC, tviC, tviE*, and *vexD* (19). CVD 908 and CVD 909 carry non-reverting site-specific chromosomal deletions in *aroC* and *aroD*, blocking the essential aromatic amino acid biosynthesis pathway (13); CVD 909 contains an additional deletion of *htrA* encoding a heat shock protease (20), and chromosomal insertion of the constitutive P_*tac*_ promoter upstream of *tviA*, resulting in constitutive expression of Vi capsular polysaccharide (12). CVD 910 and CVD 915 carry attenuating chromosomal deletions in *guaBA*, interrupting the guanine biosynthesis pathway (21); CVD 910 carries the additional deletion of *htrA* also present in CVD 909 (22). Additionally, it has been previously reported that the invasion capability for wild-type Ty2 decreases as the level of attenuation increases for a given candidate vaccine strain. Wang et al. (23), showed that viable intracellular bacteria recovered at 4 h post-invasion for CVD 909 were diminished by 2 logs below that of fully virulent wild-type Ty2. Although not published, our group have observed invasion of the less attenuated strain CVD 908 at levels between the wild-type parent Ty2 and CVD 909 *in vitro*. Indeed, clinical trials of CVD 908 reported a clinically silent “vaccinemia” in the blood of volunteers, demonstrating the invasiveness of CVD 908 *in vivo* (20), leading to the engineering of the CVD908-derived CVD 909. Moreover, after 4 h of infection, a 2 log reduction in viable intracellular bacteria was observed for CVD 915 vs. wild-type Ty2 (21).

All strains were grown on Luria-Bertani (LB) agar Lennox (Difco Laboratories, Detroit, MI). For the culture of Ty2 and Ty21a strains, bacteria were grown on solid medium without supplements. Attenuated vaccine strains were grown on solid medium supplemented as follows: (**1**) CVD 908 and CVD 909 −0.1% 2,3-Dihydroxybenzoic acid (DHB) (Sigma, St. Louis, MO), (**2**) CVD 910 and CVD 915 −1% Guanine (Sigma). After overnight incubation at 37°C, bacteria were harvested from plates, resuspended in RPMI, and diluted to obtain ~0.2 OD_600_, which for most strains of *S*. Typhi is equivalent to 10^8^ bacteria/ml (24).

### Bacterial infection

Infection was performed as described previously (8, 10). Briefly, 3-D model constructs were exposed to different bacterial strains (Table 1) at a multiplicity of infection (MOI) of 1:200 for 4 h at 37°C in RPMI (without antibiotics). The choice of the selected timepoint was a compromise between its ability to stimulate cells within the 3-D model and mimic early immune responses of resident cells (25).

### Cytokine production

Levels of interleukin (IL)-1β, IL-6, IL-8, IL-17A and tumor necrosis factor (TNF)-α cytokines were measured by using Meso Scale Discovery (MSD, Gaithersburg, MD) multiplexed-assay. Supernatants were harvested 4 h after addition of *S*. Typhi strains to the cultures and kept at −20°C until assayed. In these studies, uninfected cells (medium only) were used as negative controls. MSD was carried out following the manufacturer's instructions. The levels of sensitivity for the various cytokines measured by MSD ranged from 0.3 to 2.5 pg/ml.

### Antibodies

Cells were surface stained with anti-human antibodies (Abs) to CD11b (clone ICRF44), CD14 (clone TuK4), CD31 (clone M89D3), CD45 (clone 2D1), CD163 (clone GHI/61), IL-6 (clone MQ2-39C3), TNF-α (clone MAb11), vimentin (clone RV202) (BD Pharmingen, San Diego, CA), IL-8 (clone E8N1), CD326 (EpCAM, clone 9C4) (Biolegend, San Diego, CA), and CD3 (clone UCHT1, Beckman-Coulter, Miami, FL). Antibodies conjugated to the following fluorochromes were used in these studies: Fluorescein isothiocyanate (FITC), Phycoerythrin (PE), Peridinin chlorophyll protein (PerCP)-Cy5.5, PE-Cy7, Energy Coupled Dye or PE-Texas-Red conjugate (ECD), Pacific Blue, Brilliant Violet (BV) 570, BV605, BV650, Quantum dot (QD) 800, Alexa 647, allophycocyanin (APC)-Alexa 700 and APC-H7.

### Surface and intracellular staining

The phenotype of innate immune cells was evaluated by flow cytometry. Constructs were harvested after 4 h of exposure to *S*. Typhi strains and used to isolate single cells from the collagen-enriched matrix. Briefly, constructs were placed into a 15 ml-tube and gently washed twice with 10 ml 1X PBS (Ca^++^/Mg^++^ free). After washing, to disrupt the tissues mechanically, tissue pieces were covered with 10 mg/ml (1%) of collagen/dispase (Roche, Indianapolis, IN) and vigorously re-suspended up and down with a transfer pipette. The 15ml tube was then transferred to a 37°C 5% CO_2_ incubator for 30 min. After incubation, an 18-G needle fitted on a 5-ml syringe was used to further disrupt the construct by passing the collagenase solution through the needle 3 times and then returning the tube to the 37°C 5% CO_2_ incubator. After an additional 30 min, the pieces were again vigorously re-suspend up and down with a transfer pipette and filtered through a 40 μm filter to obtain single cells. Cells were washed with PBS 5% FCS containing Ca^++^ and Mg^++^ and used for surface and intracellular staining.

First, cells were stained with a dead-cell discriminator, violet fluorescent viability dye (ViViD, Invitrogen) (24), followed by surface staining with Abs against CD3, CD11b, CD14, CD31, CD45, CD163, CD326, and vimentin surface antigens and fixation and permeabilization with Fix & Perm cell buffers (Invitrogen, Carlsbad, CA) (26). Cells were then stained intracellularly for IL-6, IL-8, and TNF-α. Finally, cells were resuspended in fixation buffer (1% formaldehyde) and analyzed as quickly as possible by flow cytometry on an LSR-II instrument (BD Biosciences). Data were analyzed with WinList v6.0 (Verity Software House, Topsham, ME). Cells were gated based on their light scatter characteristics and specific lineage differentiation markers: CD45^+^ CD3^+^ for T cell lymphocytes, CD45^+^ CD14^+^ CD163^+^ CD11b^+^ for macrophages, CD45^−^ EpCAM^−^ CD31^−^ Vimentin^+^ for fibroblasts, CD45^−^ EpCAM^−^ CD31^+^ for endothelial cells, and CD45^−^ EpCAM^+^ for epithelial cells.

### RNA extraction

RNA isolation was performed as previously described (10). Briefly, total RNA isolation was performed using the RNeasy Mini Isolation Kit (Qiagen, Valencia, CA) and RNA quality and concentrations measured using a NanoDrop 1000 spectrophotometer.

### Human antibacterial and tight junctions RT^2^ profiler arrays

The Human Tight Junctions and Antibacterial RT^2^ Profiler PCR Arrays (Qiagen) were used to determine the expression of 168 key genes. Of these, 84 are involved in the intestinal permeability modulation and 84 are involved in innate immune response to bacteria. Arrays were performed according to the manufacturer's instructions. Briefly, DNase I -treated RNA was reverse transcribed using RT^2^ first strand Reverse Transcription Kit, and amplified material detected using RT^2^ SYBR^®;^ Green qPCR Mastermix. Real-time quantitative PCR was performed on an ABI 7900HT thermocycler (Applied Biosystems), with cycling conditions of 95°C for 10 min, 40 cycles of 95°C for 15 secs and 60°C for 1 min. Analysis of results were performed using the web-based GeneGlobe Data Analysis Center web-based Software (Qiagen). The software automatically selected an optimal set of internal control/housekeeping/ normalization genes for the analysis from the available housekeeping gene panel (i.e., ACTB, B2M, GAPDH, HPRT1, and RPLP0) on the PCR Array. The software measures and identifies the genes with the most stable expression via a non-normalized calculation. The CT values for these genes were then geometrically averaged and used for the 2(–Delta Delta Ct) calculations. The software also performs unsupervised clustergram displaying hierarchical clustering of the dataset as a heat map with dendrograms indicating co-regulated genes.

### Cell viability

The viability of the cells was assessed by quantifying lactate dehydrogenase (LDH) release into the supernatant using a commercial kit (CytoTox 96; Promega, Madison, WI). LDH is a stable cytosolic enzyme that is released into the cell culture supernatant upon cell lysis. The supernatants were harvested 4 h after addition of *S*. Typhi strains and kept at −20°C until assayed. The relative number of lysed cells was calculated by interpolating sample results into a standard curve prepared using an LDH positive control. The LDH Positive Control gives approximately the same level of enzyme found in 13,500 lysed L929 fibroblast cells.

### Statistical analysis

All statistical tests were performed using Prism software (version 5.02, GraphPad Software, La Jolla, CA). Comparisons between groups were performed using One-way ANOVA tests. Newman-Keuls Multiple Comparison tests were used as *post-hoc* tests. Correlations used the Pearson Product Moment tests. *P*-values < 0.05 were considered significant.

## Results

### Inflammatory responses after stimulation with different *S*. typhi strains

**Figure 1:**
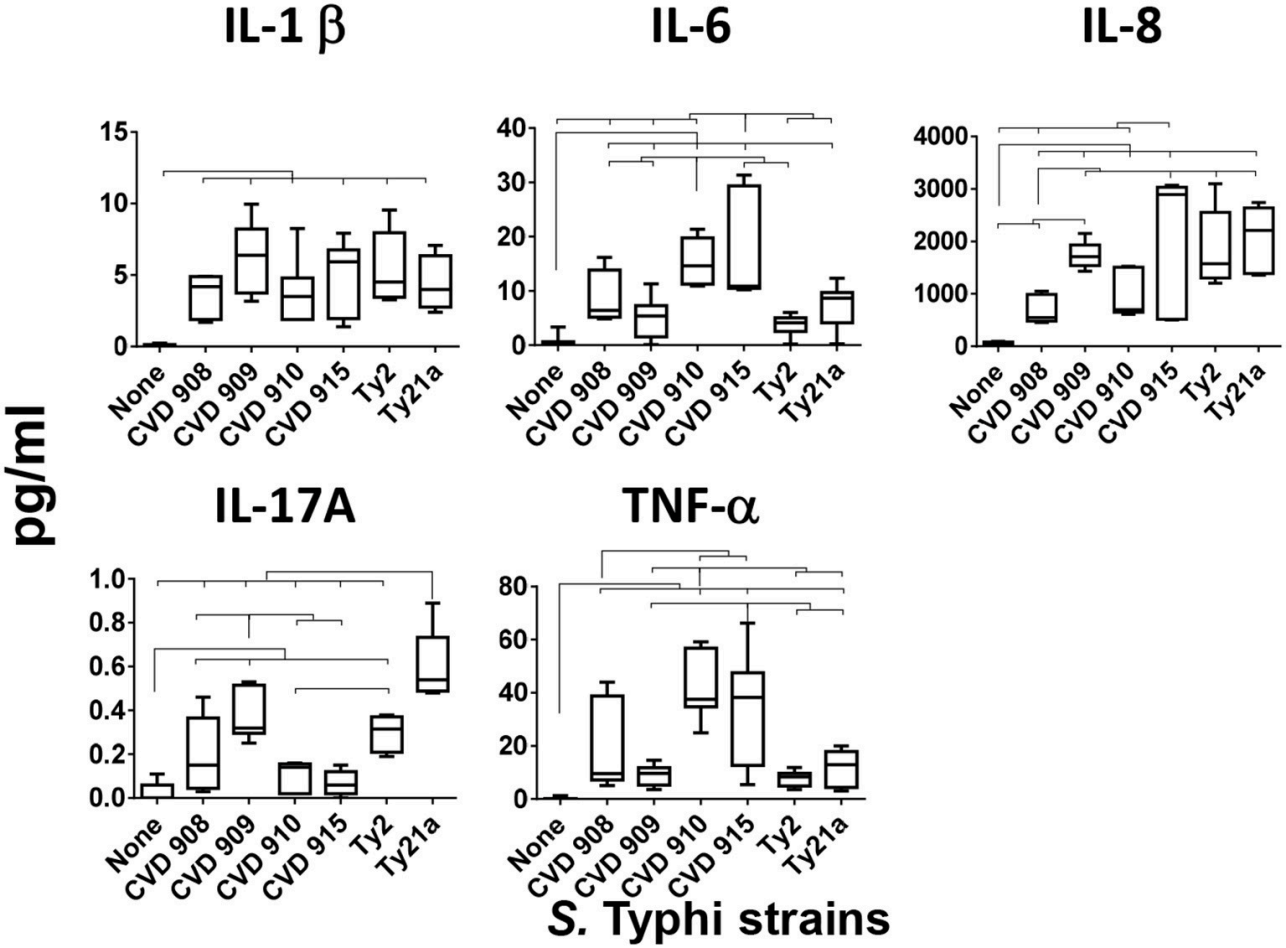
Cytokine production after stimulation with different *S*. Typhi strains. Cells from the 3-D model were untreated (none) or exposed to CVD 908, CVD 909, CVD 910, CVD 915, Ty2, or Ty21a *S*. Typhi strains. After 4 h, levels of cytokines in the cultures supernatants were measured by using the commercial Meso Scale Discovery (MSD) multiplex-assay. Bar graphs extend from the 25 to 75th percentiles, and the line in the middle represents the median of the pooled data. The whiskers delineate the smallest to the largest value. The data represent up to 6 individual experiments for each of *S*. Typhi strains with one or two replicates. Horizontal lines represent significant differences (*p* < 0.05) between the indicated culture conditions.

Because our previous studies have shown that cells from the human organotypic 3-D model can secrete pro-inflammatory cytokines after *S*. Typhi exposure (8), we examined the presence of IL-1β, IL-6, IL-8, IL-17A, and TNF-α cytokines in the supernatants of the 3-D model cultures after exposure to six different *S*. Typhi strains. Cells were left untreated (none) or exposed to either wild-type strain Ty2 or 5 *S*. Typhi vaccine/vaccine candidates derived from Ty2 (Ty21a, CVD 908, CVD 909, CVD 910, or CVD 915) (Table 1). We found that, albeit at different levels, the exposure to any of the *S*. Typhi strains resulted in increases in cytokine secretion as compared with negative controls (Figure 1). Interestingly, we observed that each *S*. Typhi strain triggered different profiles of cytokine responses by the cells composing the 3-D model. Vaccine candidate strain CVD 910 and CVD 915 promoted higher secretion of IL-6 and TNF-α than wild-type Ty2, Ty21a vaccine and vaccine candidates CVD 908 and CVD 909 (Figure 1). CVD 915 also promoted higher secretion of IL-8 as compared to two vaccine candidates, CVD 908 and CVD 910. It is also important to note that Ty21a and, to a lesser degree, Ty2 and CVD 909, were the highest inducers of IL-17A as compared to CVD 908, CVD 910, and CVD 915 (Figure 1). No difference in the induction of IL-1β was found among the strains (Figure 1).

**Figure 2:**
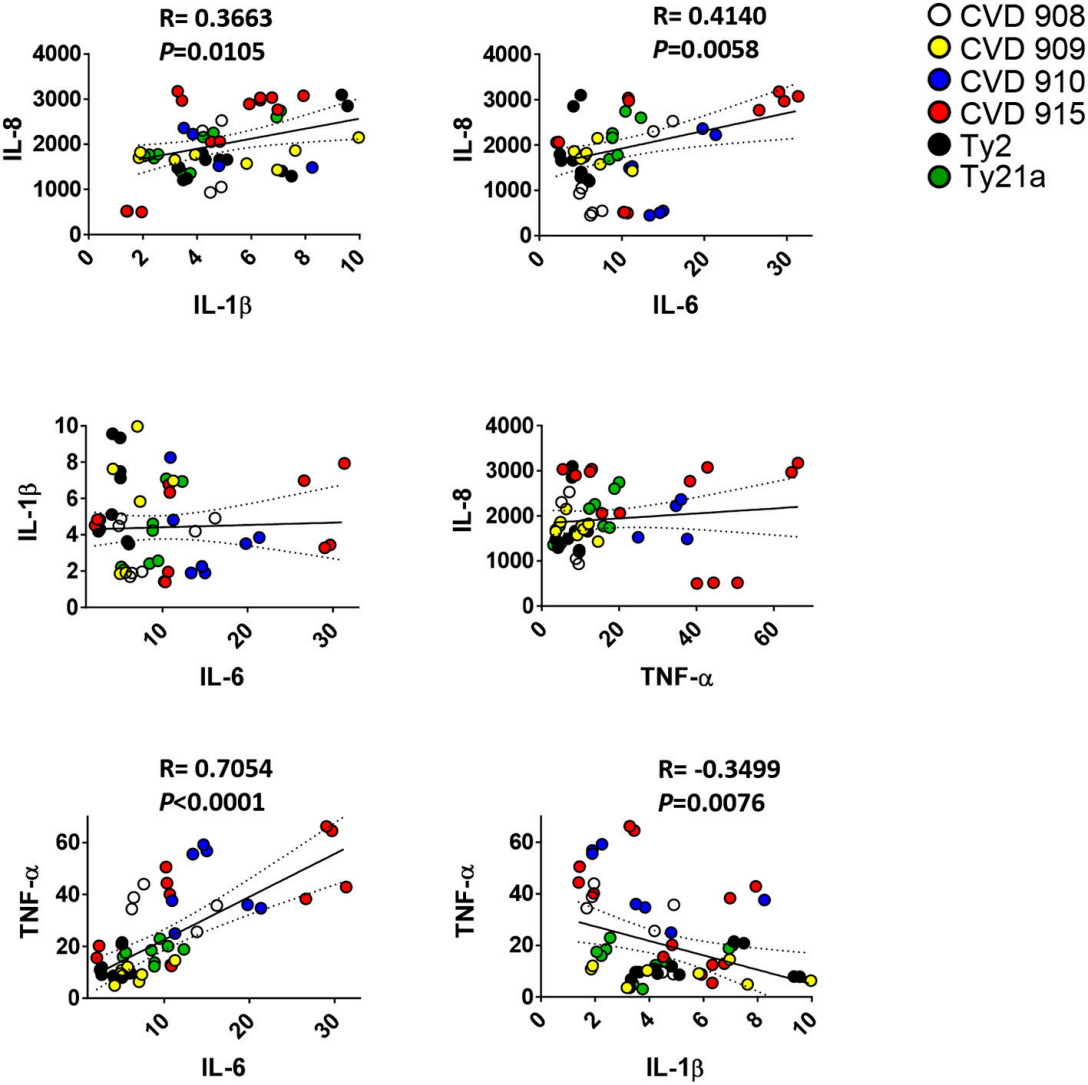
Correlation among pro-inflammatory cytokine levels. Cells from the 3-D model were untreated (none) or exposed to CVD 908, CVD 909, CVD 910, CVD 915, Ty2, or Ty21a *S*. Typhi strains. After 4 h, the levels of IL-1-β, IL-6, IL-8, and TNF-α cytokines in the cultures supernatants were measured by the commercial Meso Scale Discovery (MSD) multiplex-assay. Correlations used the Pearson Product Moment tests. The data represent up to six individual experiments for each of *S*. Typhi strains with one or two replicates. *P*-values < 0.05 were considered significant.

**Figure 3:**
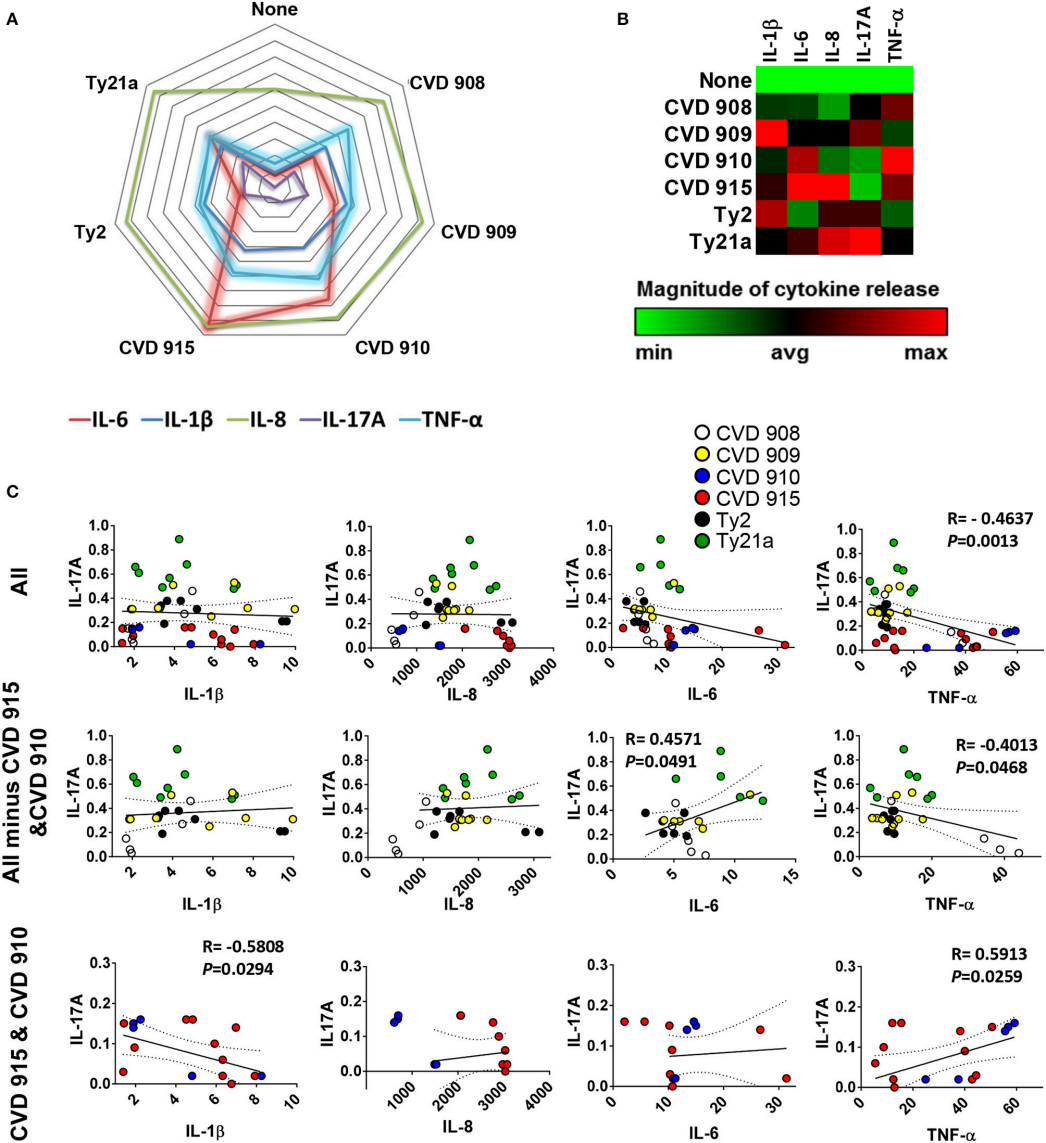
Profile of cytokine response after stimulation with different *S*. Typhi strains. Cells from the 3-D model were untreated (none) or exposed to CVD 908, CVD 909, CVD 910, CVD 915, Ty2, or Ty21a *S*. Typhi strains. After 4 h, the levels of IL-1-β, IL-6, IL-8, IL-17A, and TNF-α cytokines in the cultures supernatants were measured by the commercial Meso Scale Discovery (MSD) multiplex-assay. **(A)** Spider plot and **(B)** heat map of the mean values of the cytokines. **(C)** Correlation between IL-17A and the other pro-inflammatory cytokine levels in the supernatants from 3-D mucosa organoids after stimulation with the different *S*. Typhi strains. Correlations used the Pearson Product Moment tests. The data represent up to six individual experiments for each of *S*. Typhi strains with one or two replicates. *P*-values < 0.05 were considered significant.

We next investigated whether there was a correlation among the levels of IL-1β, IL-6, IL-8, IL-17A, and TNF-α cytokines. We observed a strong direct relationship between IL-6 and either IL-8 or TNF-α levels (Figure 2). Surprisingly, TNF-α levels were inversely correlated with IL-1β (Figure 2) and IL-17A (Figures 3A–C). Given the importance of IL-17A in mucosal immunity (27–29), we focused our subsequent analyses on IL-17A production in relation to other cytokines elicited by the various *S*. Typhi strains. No direct correlation was found between IL-17A and the others pro-inflammatory cytokines. Thus, these results suggest that IL-1β, IL-6, IL-8, and TNF-α cytokines have no additive or synergistic influence on IL-17A behavior. To test this hypothesis, we divided the results of IL-17A into two sub-groups: (**1**) IL-17A responses triggered by all strains minus the responses triggered by CVD 910 and CVD 915, and (**2**) IL-17A responses triggered only by CVD 910 and CVD 915 strains. CVD 910 and CVD 915 strains prompted the lowest production of IL-17A as compared to the other strains (Figures 3A,B). CVD 910 and CVD 915 are also the only vaccine candidate strains that carry *guaBA* deletion. The attenuation of CVD 908 and CVD 909 vaccine candidate strains is based on the deletions of *aroC, aroD* genes. We found that although IL-17A levels inversely correlated with TNF-α production in the group lacking cytokine responses induced by CVD 910 and CVD 915, it directly correlated with TNF-α production in the group containing cytokines responses induced by only CVD 910 and CVD 915 (Figure 3C). We also found that IL-17A levels inversely correlated with IL-1β levels in the group containing cytokines responses induced by only CVD 910 and CVD 915. Finally, we found that IL-17A levels directly associated with IL-6 levels in the group lacking cytokines responses induced by CVD 910 and CVD 915. Thus, strains with different gene mutations might contribute by different mechanisms to inflammation.

**Figure 4:**
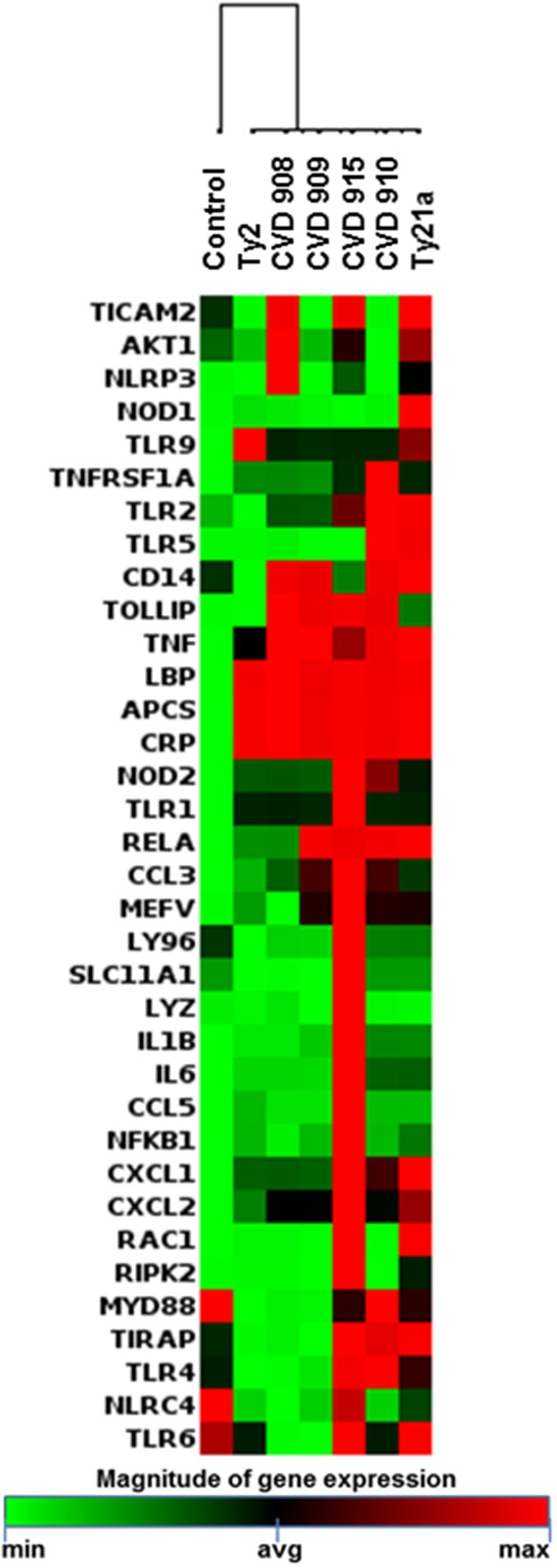
Inflammatory Responses after stimulation with different S. Typhi strains. Cells from the 3-D model were untreated (none) or exposed to CVD 908, CVD 909, CVD 910, CVD 915, Ty2, or Ty21a *S*. Typhi strains. After 4 h, tissues were collected and used to measure the gene expression by qRT-PCR. Data shown are unsupervised clustergram displaying hierarchical clustering of the genes related to the inflammatory response signaling as a heat map as a heat map. Geometric mean values of two independent experiments with two replicates. Results are shown as x-fold regulation relative to the control group. Genes detected by the Antimicrobial Responses RT^2^ Profiler PCR Array.

To identify the key molecular cluster(s) characteristic of the inflammatory pathway responses against the different *S*. Typhi strains, expression of inflammatory genes was evaluated by qRT-PCR (Supplemental Table 1; Figure 4). By performing unsupervised cluster analysis, we observed roughly two clusters representing low and high expression of inflammatory genes, one composed by genes activated by Ty2, CVD 908, and CVD 909, and another one by genes activated by CVD 910, CVD 915 and Ty21a strains, respectively (Figure 4).

### IL-23A and caspase-1 expression after stimulation with different *S*. typhi strains

**Figure 5:**
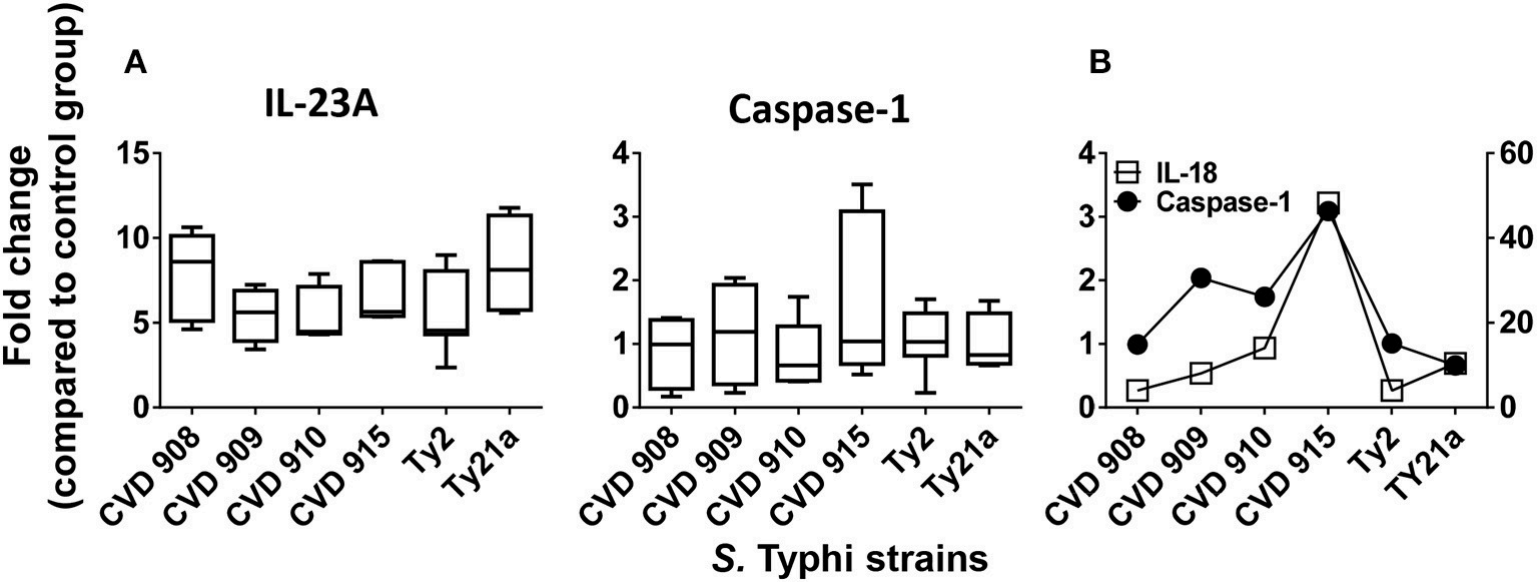
IL-23A and inflammasome-caspase-1 pathway after stimulation with different *S*. Typhi strains. Cells from the 3-D model were untreated (none) or exposed to CVD 908, CVD 909, CVD 910, CVD 915, Ty2, or Ty21a *S*. Typhi strains. After 4 h, tissues were collected and used to measure gene expression by qRT-PCR. **(A)** IL-23A and caspase-1 gene expression by qRT-PCR. Bar graphs extend from the 25 to 75th percentiles, and the line in the middle represents the median of the pooled data. The whiskers delineate the smallest to the largest value. The data represent up to six individual experiments for each of *S*. Typhi strains with one or two replicates. **(B)** A representative experiment showing the concomitant expression of IL-18 and Caspase-1.

Since IL-17 synthesis is induced by IL-23 (30) and IL-17-induced effect can use inflammasome-caspase-1 pathway (31), we next investigated changes in the expression of IL-23A and caspase-1 after stimulation with the 6 different *S*. Typhi strains by qRT-PCR. We found that, although not statistically different, IL-23A expression exhibited a trend to be higher in cultures exposed to CVD 908 and Ty21a as compared to CVD 909, CVD 910, CVD 915 and Ty2 strains (Figure 5A). Interestingly, distinct increases of caspase-1 expression were observed after CVD 915 exposure. We next investigated the relationship between caspase-1 and IL-18 expression. Caspase-1 activation is known to result in the processing and secretion of the IL-1β and IL-18 proinflammatory cytokines (32), a mechanism called inflammasome. We found that exposure to CVD915 resulted in the higher concomitant expression of caspase-1 and IL-18 as compared to other *S*. Typhi strains (Figure 5B). These results are in agreement with the results obtained for cytokine secretion, where exposure to CVD 915 resulted in the highest production of inflammasome-mediated TNF-α and IL-6 cytokines.

### Effect of the different *Salmonella* strains on antibacterial gene signatures

**Figure 6:**
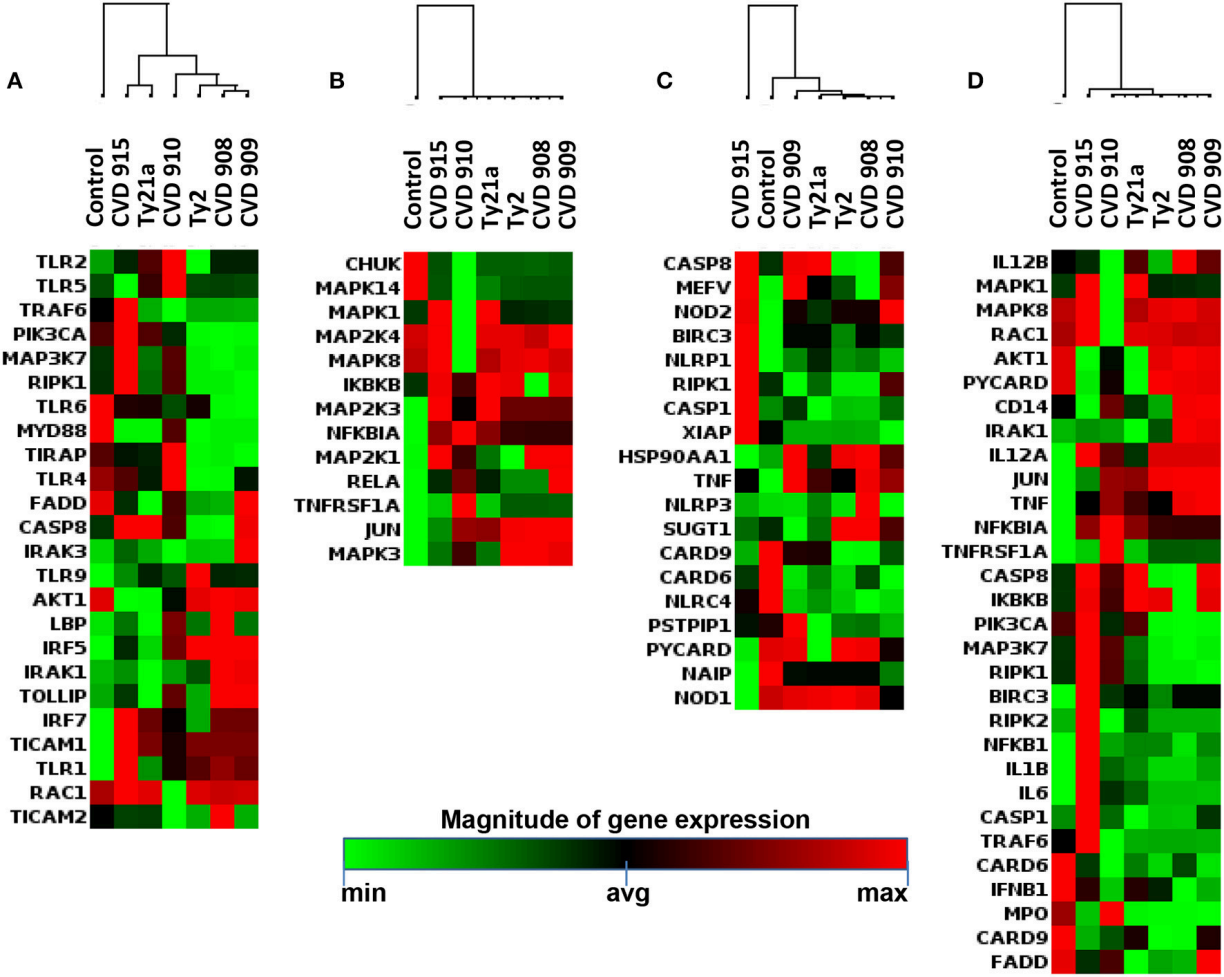
Antimicrobial Responses after stimulation with different *S*. Typhi strains. Cells from the 3-D model were untreated (none) or exposed to CVD 908, CVD 909, CVD 910, CVD 915, Ty2, or Ty21a *S*. Typhi strains. After 4 h, tissues were collected and used to measure gene expression by qRT-PCR. Data shown are unsupervised clustergram displaying hierarchical clustering of the dataset as a heat map. Geometric mean values of two independent experiments with two replicates. Results are shown as x-fold regulation relative to the control group. Genes detected by the Antimicrobial Responses RT^2^ Profiler PCR Array: **(A)** Toll-Like Receptor (TLR) signaling. **(B)** Downstream signaling of antibacterial responses. **(C)** NOD-Like Receptor (NLR) signaling. **(D)** Apoptosis signaling.

**Figure 7:**
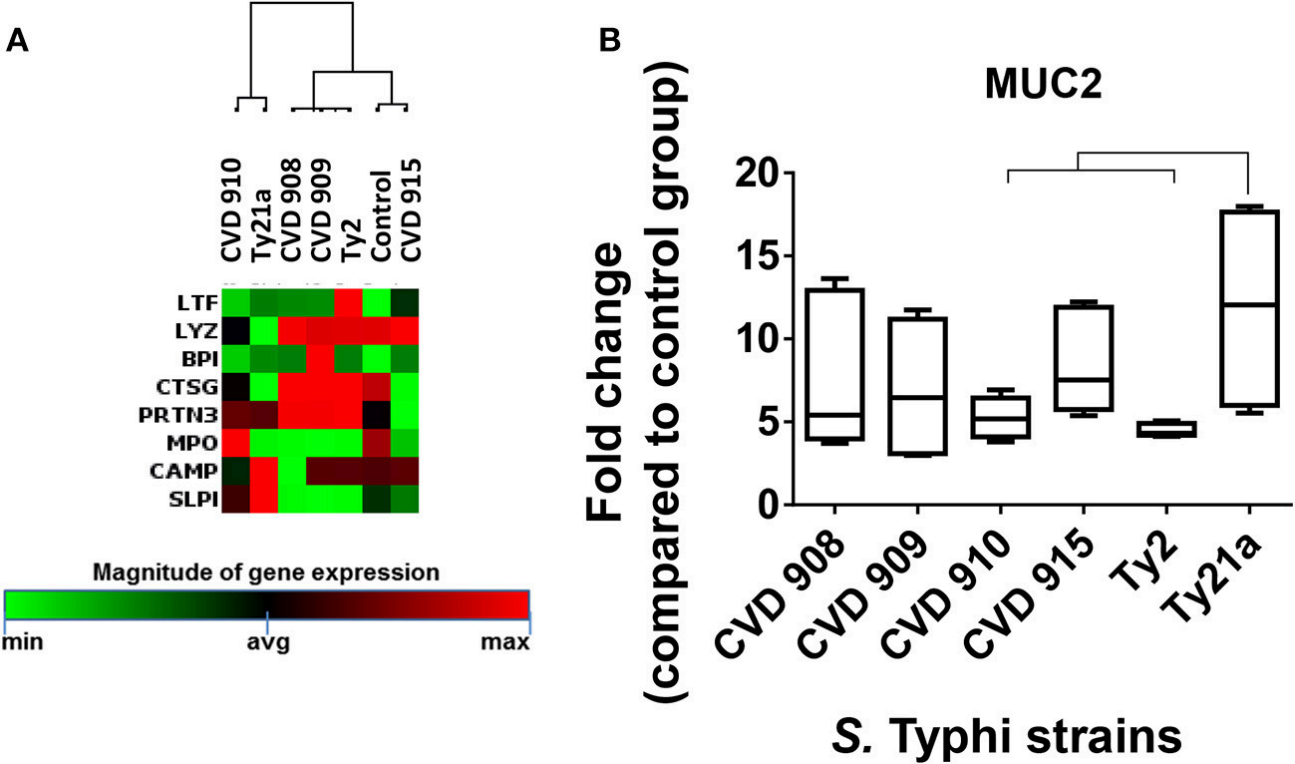
Differential expression of mucus and antimicrobial peptide signaling after stimulation with different *S*. Typhi strains. Cells from the 3-D model were untreated (none) or exposed to CVD 908, CVD 909, CVD 910, CVD 915, Ty2, or Ty21a *S*. Typhi strains. After 4 h, tissues were collected and used to measure the gene expression by qRT-PCR. **(A)** Data shown are unsupervised clustergram displaying hierarchical clustering of the genes related to the antimicrobial signaling as a heat map. Geometric mean values of two independent experiments with two replicates. Results are shown as x-fold regulation relative to the control group. Genes detected by the Antibacterial Response RT^2^ Profiler PCR Array. **(B)** Mucus (MUC2) gene expression. Bar graphs extend from the 25 to 75th percentiles, and the line in the middle represents the median of the pooled data. The whiskers delineate the smallest to the largest value. The data represent six individual experiments with one or two replicates. Horizontal lines represent significant differences (*p* < 0.05) between the indicated culture conditions.

To determine whether the inflammatory effects described above result in specific antibacterial gene signatures by the *S*. Typhi strains, we next investigated *S*. Typhi strain regulation of the genes related to Toll-Like Receptor (TLR), downstream antibacterial responses, NOD-Like Receptor (NLR), apoptosis, and antimicrobial peptide signaling by qRT-PCR (Supplemental Table 1; Figure 6). Similar to the observations for inflammatory genes, the hierarchical clustering of TLR, downstream antibacterial responses, and apoptosis genes showed roughly two distinct clusters, one composed by genes activated by Ty2, CVD 908, and CVD 909, and another one formed by genes activated by CVD 910, CVD 915 and Ty21a strains (Figures 6A,B,D). Surprisingly, clustering of NLR and antimicrobial peptide genes revealed that CVD 915 clustered separately from the other *S*. Typhi strains (Figures 6C, 7A). It is important to note that NLR signaling includes inflammasome genes, such as caspase-1.

As described previously (8, 10), epithelial cells in our model can differentiate into mucus-secreting goblet cells. In fact, the mucus layer operates together with other antimicrobial peptides to restrain bacteria to the intestinal lumen (33). Thus, we next examined the expression MUC-2, a mucin encompassed in intestinal mucus layer, by qRT-PCR. While exposure to any *S*. Typhi strains resulted in increases in MUC-2 expression, significantly higher expression of MUC-2 was observed after exposure to Ty21a as compared to CVD 910 and Ty2 strains (Figure 7B). Thus, gene clustering provide important clues regarding the similarities and differences of the activation of various pathways depending on strain construction.

### Evaluation of the immune cell responses after exposure to *S*. typhi

**Figure 8:**
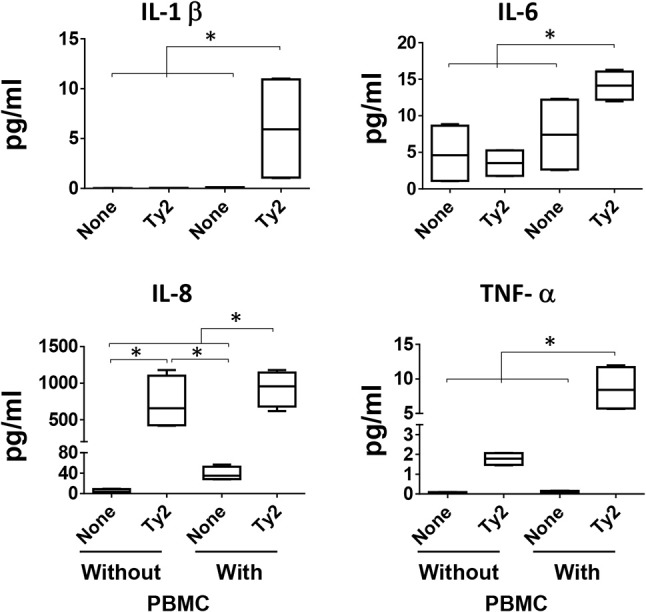
Impact of PBMC on cytokine production. During the building of the 3-D model, immune cells (i.e., PBMC) were added, or not, to the culture using a “minus/plus” one-factor approach. Once the 3-D models reached the maturity (~17 days after initiation of the culture), the models were exposed or not to *S*. Typhi Ty2 strain. After 4 h, cell-free supernatants were collected and used to measure IL-1β, IL-6, IL-8, and TNF-α secretion by MSD. Bar graphs extend from the 25 to 75th percentiles, and the line in the middle represents the median of the pooled data. The whiskers delineate the smallest to the largest value. Data are representative of two independent experiments with two replicates. *p < 0.05, Horizontal lines represent significant differences between the indicated culture conditions.

It is well-known that IL-1β, IL-6, IL-8, and TNF-α cytokines are mainly produced by immune cells after insults to the intestinal mucosa (34). Thus, since all previous experiments were performed using 3-D models containing immune cells, we next performed experimental “gain and loss” studies to define the importance of these cells (i.e., lymphocytes and macrophages) (PBMC) in the production of the cytokines. Specifically, during the building of the 3-D model, lymphocytes/monocytes rich PBMC were added, or not, to the culture using a “minus/plus” one-factor approach. Once the 3-D model reached maturity (~17 days after initiation of the culture), the model was exposed, or not, to the Ty2 strain. After 4 h of stimulation, supernatants were collected and used to measure IL-1β, IL-6, IL-8, and TNF-α cytokine secretion. We observed that regardless of the bacteria strain, IL-1β, IL-6, and TNF-α secretion was higher in the supernatants from models that contained immune cells (i.e., PBMC) as compared to 3-D models built without PBMC (Figure 8). No significant differences in IL-8 secretion were observed in the presence or absence of immune cells. Thus, immune cells play an essential role in the induction of IL-1β, IL-6, and TNF-α cytokines.

**Figure 9:**
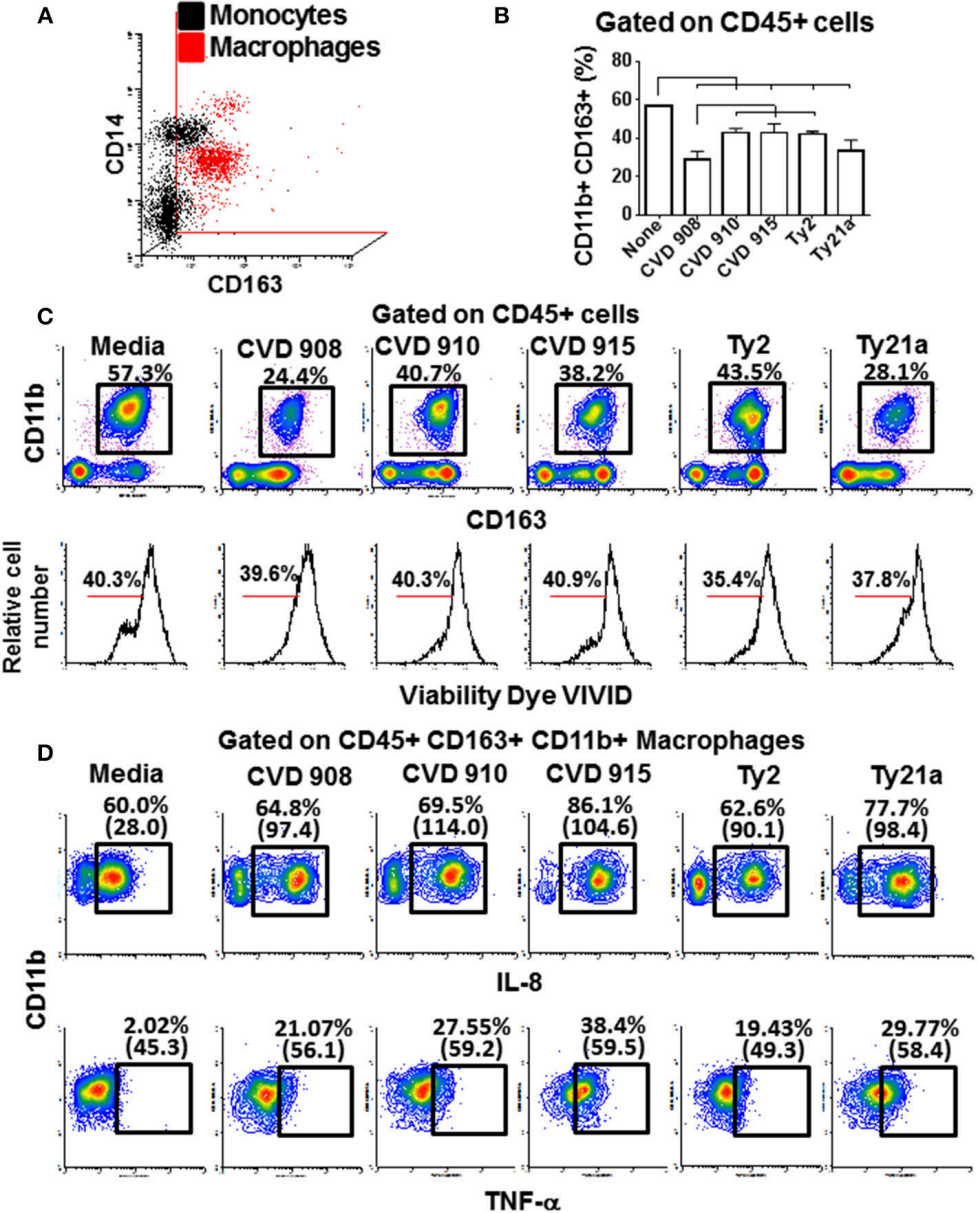
Macrophage phenotype, migration and production of cytokine after stimulation with different *S*. Typhi strains. The 3-D model was left untreated (media) or exposed to CVD 908, CVD 910, CVD 915, Ty2, or Ty21a *S*. Typhi strains. After 4 h, tissues were collected and disaggregated and used to perform flow cytometry. **(A)** Upper panel, comparison between macrophages present in the 3-D model and monocytes present in the blood. Lower panel, macrophage vialitity detected using violet fluorescent dye ViViD. **(B)** Levels of resident macrophages in various culture conditions. Horizontal lines represent significant differences (*p* < 0.05) between the indicated culture conditions. **(C)** Representative experiment showing the levels of resident macrophages present in the 3-D tissues. **(D)** IL-8 and TNF-α intracellular staining. Numbers between in parentheses represent mean fluorescence intensity.

These findings led us to question the role of macrophages and lymphocytes in the secretion of these cytokines. Macrophages have an essential role fighting against *S*. Typhi infection (4, 35). After 4 h of infection, tissues from the 3-D model were collected and disaggregated by collagenase and used to perform flow cytometry. To evaluate the phenotype and function of the macrophages, isolated cells were stained for surface (CD11b, CD14, CD45, and CD163) and intracellular (IL-6, IL-8, and TNF-α) markers. When compared to monocytes present in blood, macrophages from the 3-D model were CD14 low among a mainly CD163 population (Figure 9A), a phenotype characteristic of resident macrophages (36). As shown in Figure 9B,C, lower numbers of macrophages were present in the 3-D model tissues when exposed to *S*. Typhi strains as compared with the controls cultures with media only. Cultures exposed to the CVD 908 strain had the higher decrease in macrophages as compared to the other *S*. Typhi strains. Because the cell viability of macrophages in these cultures was similar to the controls (media, Figure 9C lower panel), one can hypothesize that the macrophages migrated from the matrix (equivalent to the mucosa *in vivo*) to the site of infection (i.e., into the supernatants). However, one cannot exclude the possibility that the amino dyes used to detect the viability of the cells were not sensitive to identify early apoptosis of the dying macrophage after *Salmonella* exposure. Finally, IL-8 and TNF-α expression was higher both per cell (mean fluorescence intensity), and per frequency in the tissues exposed to *S*. Typhi strains as compared to the controls (Figure 9D). Culture exposed to CVD 915 strain had greater increases in IL-8 and TNF-α expression as compared to the other strains. Absent or marginal increases of IL-6 expression were also observed (data not shown). Of note, no increases in the production of IL-6, IL-8, and TNF-α cytokines by CD45^+^ CD3^+^ for T cell lymphocytes were observed (data not shown), confirming that these cytokines were mainly produced by macrophages. Thus, after exposure to *S*. Typhi, immune cells from the 3-D model are activated and produced cytokines. Macrophages had a sizable role in these responses and underwent phenotypic and functional changes that depended on the *S*. Typhi strain encountered.

### Activation of intestinal epithelial and stromal cells

**Figure 10:**
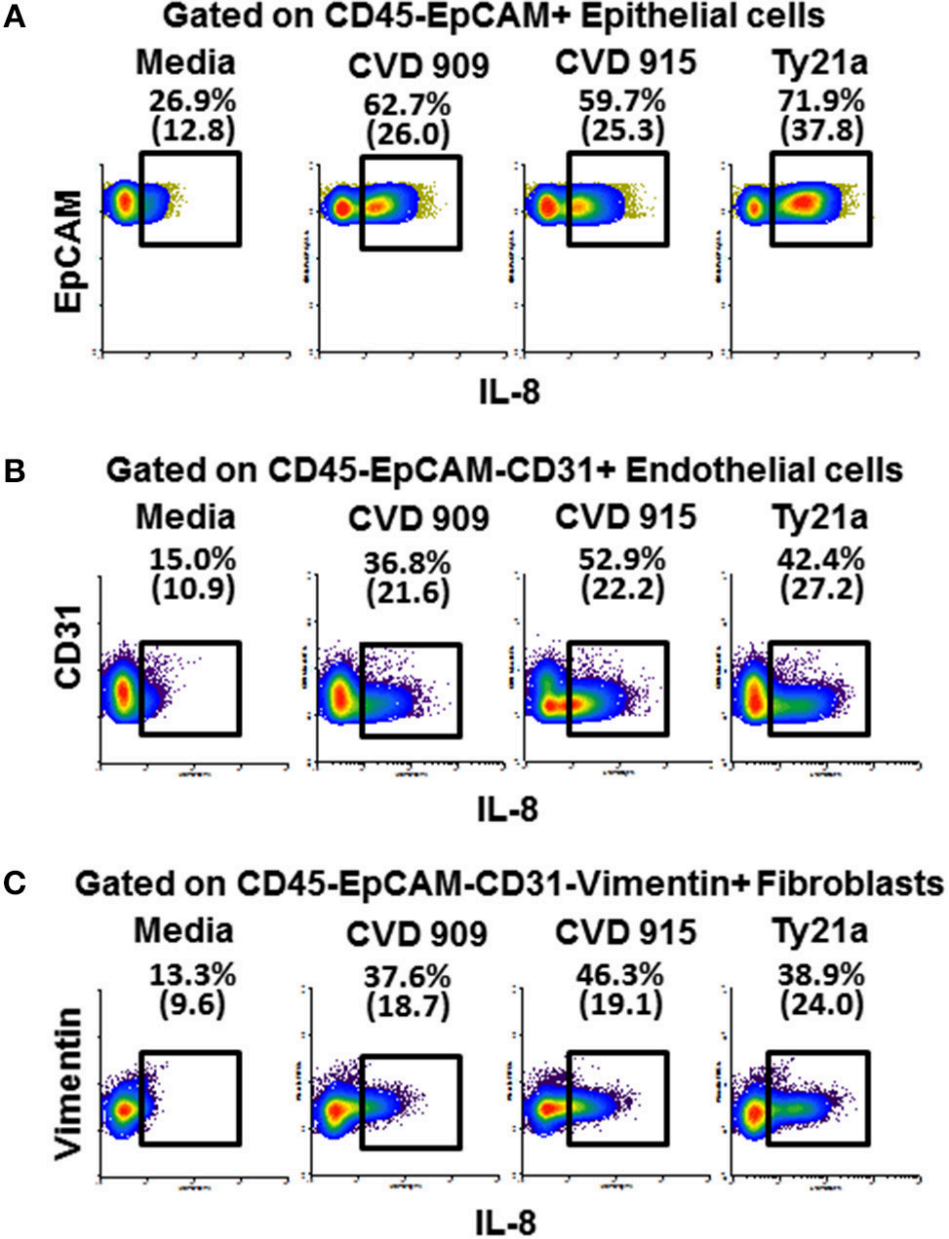
Expression of IL-8 by epithelial and stromal cells after stimulation with different *S*. Typhi strains. The 3-D model was left untreated (media) or exposed to CVD 909, CVD 915, or Ty21a *S*. Typhi strains. After 4 h, tissues were collected and disaggregated and used to perform IL-8 intracellular staining and flow cytometric analysis. Cells were gated based on their scatter characteristics and specific lineage differentiation markers: **(A)** CD45-EpCAM+ for epithelial cells, **(B)** CD45-EpCAM-CD31+ for endothelial cells, and **(C)** CD45-EpCAM-CD31-Vimentin+ for fibroblasts. Numbers in parentheses represent mean fluorescence intensity.

Because after *S*. Typhi exposure we observed substantial increases of IL-8 in the absence of immune cells, and IL-8 is not only produced by immune cells but also by stromal cells (37), we next measured the IL-8 expression in fibroblasts, endothelial and epithelial cells. After 4 h of exposure to either CVD 909, CVD 915, or Ty21a, tissues were disaggregated and used to measure IL-8 intracellular staining and flow cytometry analysis. As expected, after *S*. Typhi exposure we observed substantial increases of IL-8 expression in fibroblasts, endothelial and epithelial cells (Figures 10A–C). We also observed that CVD 915 triggered the higher expression of IL-8 by fibroblasts and endothelial cells as compared to other *S*. Typhi strains (Figures 10B,C).

**Figure 11:**
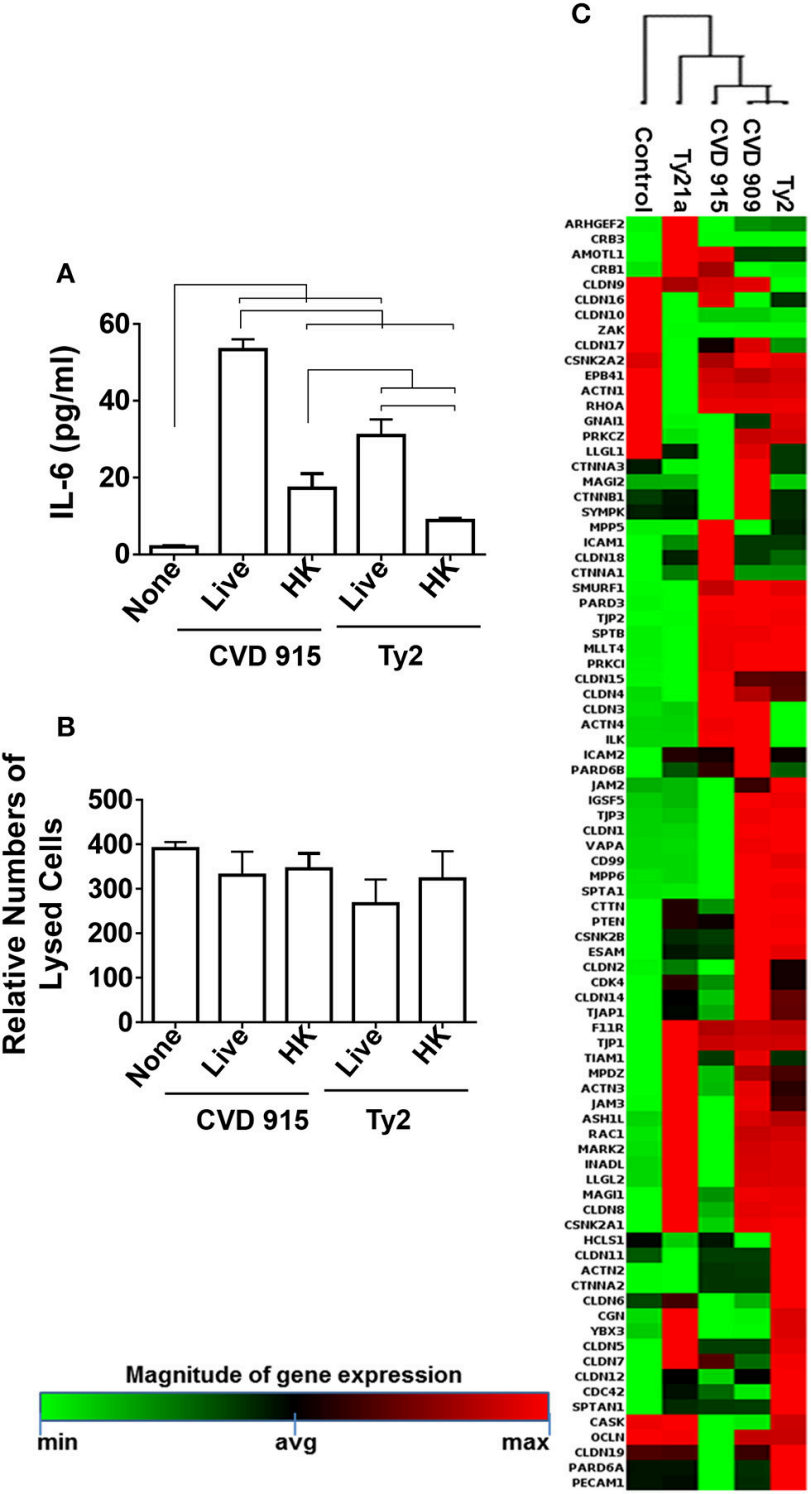
Effect of *S*. Typhi exposure on the epithelial cell barrier. Cells from the 3-D model were untreated (none) or exposed to live or heat-killed (HK) *S*. Typhi strain CVD 915 or Ty2. **(A)** After 4 h, levels of IL-6 in the cultures supernatants were measured by Meso Scale Discovery (MSD) assay. Bars represent mean ± SE. Horizontal lines represent significant differences (*p* < 0.05) between the indicated culture conditions. **(B)** The cell viability was also measured in supernatants by commercial LDH assay. Bars represent means ± SE of 1 experiment with three replicates. **(C)** Cells from the 3-D model were untreated (none) or exposed to CVD 909, CVD 915, Ty2, or Ty21a *S*. Typhi strains. After 4 h, tissues were collected and used to measure the gene expression by qRT-PCR. Data shown represents an unsupervised clustergram displaying hierarchical clustering of the dataset as a heat map. Geometric mean values of two independent experiments with two replicates. Results are shown as x-fold regulation relative to the control group. Genes expression was detected by using the Tight Junctions RT^2^ Profiler PCR Array.

Since *in vitro* CVD 915 has a poor multiplication capability when cultured in tissue medium (Table 1) as compared to Ty2, we hypothesized that host responses to CVD 915 might be related, at least in part, to the inability of bacteria to multiply. To evaluate this possibility, we exposed the 3D-model to either CVD 915 or Ty2 strains. We used as a control, heat-killed bacteria. After 4 h of incubation, the levels of IL-6 in the culture supernatants were measured by MSD. Regardless of whether CVD 915 was live or heat-killed, CVD 915 induced higher IL-6 secretion as compared to Ty2 (Figure 11A). Thus, the production of IL-6 appears to be unrelated to the bacteria multiplication capability. We next evaluated the effect of the bacteria infection on cell viability. Cell viability was evaluated by LDH, a stable cytosolic enzyme that is released into cell supernatant upon cell lysis. After 4 h in the presence of live or heat-killed CVD 915 or Ty2, we observed comparable levels of cell death with the different culture conditions (Figure 11B). Thus, the differential CVD915 effect observed over other *S*. Typhi strains on host responses appear to be unrelated to its multiplication capabilities or induction of host cell killing.

### Intestinal epithelial barrier integrity

Because both inflammation and changes in the mucus layer are well-known to induce modifications in the epithelial barrier (38–40), we next studied the effect of *S*. Typhi strains on tight junctions genes (e.g., those responsible for the maintenance of epithelial cell barrier integrity) by qRT-PCR (Supplemental Table 2; Figure 11C). As described above for antibacterial gene clustering, hierarchical clustering of tight junctions genes showed two distinct clusters, one composed by genes activated by Ty21a and CVD 915, and another one characterized by genes activated by CVD 909 and Ty2 strains (Figure 11C). Thus, tight junctions gene clustering further confirm the similarity of the pathways activated by the strain construction and divided them into two groups: one composed of strains carrying *guaBA* (e.g., CVD 915) deletion, and another composed of strains carrying *aroC, aroD* deletions (e.g., CVD 909). Strains carrying *guaBA* appear to behave similarly to the Ty21a vaccine strain, while strains carrying *aroC, aroD* deletions appear to be more closely related to the wild-type Ty2 strain.

## Discussion

Although immunity induced by typhoid fever is of moderate magnitude and short-lived, and provides very limited protection from re-infection, the oral attenuated typhoid vaccine Ty21a generates protection rates reaching up to 92% which lasts for several years (4, 5). Thus, it is reasonable to speculate that there might be important differences on how wild-type *Salmonella* and attenuated typhoid vaccine strains stimulate host immune responses. It is important to note that CVD 908, CVD 909, CVD 910, and CVD 915 vaccine candidates used in this manuscript all have native virulence factors intact; attenuating mechanisms are directly related to specific chromosomal deletions of genes affecting metabolic functions, which in turn will limit the number of bacterial divisions *in vivo*. These strains were engineered to maximize immunogenicity and protective efficacy while minimizing clinical reactogenicity that typically accompany strong inflammatory responses. We found that genetic engineering of *S*. Typhi vaccine strains elicits changes in a model that closely resembles the environment of the human gut, not only in intestinal permeability and secretion of inflammatory cytokines, but also in the phenotype and activation pathways elicited in cells of the innate immune system. Thus, the results presented in this manuscript represent one-step forward in the understanding of how the regulation of *S*. Typhi gene expression dictates the outcome of oral vaccines.

Based on hierarchical clustering of antibacterial and tight junctions gene expression, we could roughly divide the vaccine candidates into two distinct clusters: one composed of strains carrying *guaBA* deletions (e.g., CVD 910 and CVD 915), and another composed of strains carrying *aroC, aroD* deletions (e.g., CVD 908 and CVD 909). Strains carrying *guaBA* appear to be closely related to the Ty21a vaccine strain, while strains carrying *aroC, aroD* deletions seem to be closely associated to the wild-type Ty2 strain. Surprisingly, strains carrying *guaBA* deletions (e.g., CVD 910 and CVD 915) were more prone to induce inflammatory responses than strains carrying *aroC, aroD* deletions (e.g., CVD 908 and CVD 909) which were generally observed to elicit lower inflammatory responses. Another striking observation is that strains carrying *guaBA* deletions elicited marked increases on IL-6 responses when compared to strains carrying *aroC, aroD* deletions. It is well-known that IL-6 plays an important role in the preservation of intestinal permeability by repairing epithelial layer after injury (41–44). Thus, it is tempting to speculate that strains carrying *guaBA* deletions would have a milder effect on the epithelial layer. Supporting this hypothesis is the fact that CVD 915 had high similarity to Ty21a and promoted a more moderate up-regulation of tight junctions genes as compared to CVD 909 and Ty2 strains. Moreover, exposure to CVD 915, and to a lesser extent CVD 910, resulted in higher inflammasome activation (i.e., concomitant expression of caspase-1 and IL-18) as compared to the other *S*. Typhi strains. Inflammasome activation is a critical step by which the innate immune system recognizes and limits bacterial infection (32). Indeed, inflammasome activation during *Salmonella* infection has been shown to result in protective host responses (45).

In contrast, strains carrying *aroC, aroD* deletions (e.g., CVD 908 and CVD 909) were more predisposed to induce secretion of the IL-17A cytokine than the strains carrying *guaBA* deletion (*e.g*., CVD 910 and CVD 915). We found that, although IL-17A levels inversely correlated with TNF-α production in the group carrying *aroC, aroD* deletions (e.g., CVD 908 and CVD 909), it directly correlated with TNF-α production in the group carrying *guaBA* deletions (e.g., CVD 910 and CVD 915). Since, IL-17 has been shown to act in synergy with TNF-α to induce endothelial cell activation to sustain and enhance neutrophil influx to sites of inflammation (46), it is possible that strains carrying *guaBA* deletions will promote a more robust phagocytic infiltration. This hypothesis is supported by our results showing that strains carrying *guaBA* deletions trigger higher expression of neutrophil-attracting chemokines CXCL1 and CXCL2 that facilitate diapedesis (37) than strains carrying *aroC, aroD* deletions. Also, IL-17 in conjunction with IL-23 have a fundamental role in the acute response to infection (47). Specifically, IL-23 regulates human B-cell immune responses (48). Here, we found that IL-23 expression was higher in cultures exposed to CVD 908 and Ty21a as compared to CVD 909, CVD 910, CVD 915, and Ty2 strains. While in clinical trials both CVD 909 and CVD 908 were highly immunogenic, inducing humoral and cellular-mediated immune (CMI) responses, CVD 908 was found to prompt higher levels of *Salmonella*-specific antibodies than CVD 909 (11–13). Thus, it is reasonable to expect that strains carrying *guaBA* deletions will induce lower humoral responses than Ty21a strain.

In conclusion, our data support the hypothesis that the nature of the specific attenuating mutations used in the construction of live oral *S*. Typhi-based candidate vaccines can be linked to defined immunological pathways elicited after immunization, which may in turn affect the level of intestinal epithelial cell stimulation, the profiles of innate immune cells activated, and the characteristics of the ensuing adaptive response. Our data clearly illustrate this point when comparing cytokine responses of cells in the 3-D model after infection with vaccines carrying deletions in the *guaBA* locus (affecting DNA synthesis pathway) vs. vaccines carrying deletions in *aroC* and *aroD* (affecting synthesis of aromatic amino acids). This dependence of vaccine-induced immunity on the types of genes deleted from a target pathogen has fundamental implications in vaccine development. For example, when prioritizing the type of immune responses, humoral vs. CMI, to target with specific attenuating mutations, an ideal vaccine strain must contain deletions driving immune responses believed to be associated with protection, such responses be known. Finally, with further refining of the technology, we suggest that 3-D models may prove to be exceptionally useful in the rational design of live attenuated vaccines with engineered immunological characteristics as compared to vaccines which have been designed using largely empirical approaches.

## Author contributions

JG and RS-G designed the study, performed the experiments, analyzed the data and wrote the manuscript; ML, AF and MS contributed to the design and analysis of the data and edited and reviewed the manuscript.

### Conflict of interest statement

The authors declare that the research was conducted in the absence of any commercial or financial relationships that could be construed as a potential conflict of interest.

## Abbreviations

MSD: meso scale discovery multiplexed assay
3-D: three-dimensional
*S*. Typhi: *Salmonella enterica* serovar Typhi
IL: interleukin
TNF: tumor necrosis factor
RWV: rotating wall vessels
