# Supplementary material for: Manipulation of Salmonella Typhi Gene Expression Impacts Innate Cell Responses in the Human Intestinal Mucosa
Source: Front Immunol. 2018 Nov 1;9:2543. doi: 10.3389/fimmu.2018.02543 (PMC6221971; doi:10.3389/fimmu.2018.02543)
Supplement: Supplementary file 1 [file Data_Sheet_1.PDF]

**Supplemental Table 1. List of the Genes detected by the Antibacterial Response RT$^2$ Profiler PCR Array**

| Description | | Symbol | Description | Refseq |
|---|---|---|---|---|
| Toll-Like Receptor (TLR) Signaling | Toll-Like Receptors & Cofactors | Cd14 | CD14 antigen | NM_009841 |
| | | LY96 (MD-2) | Lymphocyte antigen 96 | NM_016923 |
| | | Tlr1 | Toll-like receptor 1 | NM_030682 |
| | | Tlr2 | Toll-like receptor 2 | NM_011905 |
| | | Tlr4 | Toll-like receptor 4 | NM_021297 |
| | | Tlr5 | Toll-like receptor 5 | NM_016928 |
| | | Tlr6 | Toll-like receptor 6 | NM_011604 |
| | | Tlr9 | Toll-like receptor 9 | NM_031178 |
| | MYD88-Dependent: | Fadd | Fas (TNFRSF6)-associated via death domain | NM_010175 |
| | | Irak1 | Interleukin-1 receptor-associated kinase 1 | NM_008363 |
| | | Irak3 | Interleukin-1 receptor-associated kinase 3 | NM_028679 |
| | | Irf5 | Interferon regulatory factor 5 | NM_012057 |
| | | Irf7 | Interferon regulatory factor 7 | NM_016850 |
| | | MAP3K7 (TAK1) | Mitogen-activated protein kinase kinase kinase 7 | NM_172688 |
| | | Myd88 | Myeloid differentiation primary response gene 88 | NM_010851 |
| | | Tirap | Toll-interleukin 1 receptor (TIR) domain-containing adaptor protein | NM_054096 |
| | | Tlr1 | Toll-like receptor 1 | NM_030682 |
| | | Tlr2 | Toll-like receptor 2 | NM_011905 |
| | | Tlr4 | Toll-like receptor 4 | NM_021297 |
| | | Tlr5 | Toll-like receptor 5 | NM_016928 |
| | | Tlr6 | Toll-like receptor 6 | NM_011604 |
| | | Tlr9 | Toll-like receptor 9 | NM_031178 |
| | | Tollip | Toll interacting protein | NM_023764 |
| | | Traf6 | Tnf receptor-associated factor 6 | NM_009424 |
| | TICAM1 (TRIF)-Dependent Signaling (MYD88-Independent) | Irf5 | Interferon regulatory factor 5 | NM_012057 |
| | | Irf7 | Interferon regulatory factor 7 | NM_016850 |
| | | MAP3K7 (TAK1) | Mitogen-activated protein kinase kinase kinase 7 | NM_172688 |
| | | Ripk1 | Receptor (TNFRSF)-interacting serine-threonine kinase 1 | NM_009068 |
| | | TICAM1 (TRIF) | Toll-like receptor adaptor molecule 1 | NM_174989 |
| | | TICAM2 (TRAM) | Toll-like receptor adaptor molecule 2 | NM_173394 |
| | | Tlr4 | Toll-like receptor 4 | NM_021297 |
| | | Traf6 | Tnf receptor-associated factor 6 | NM_009424 |
| | Other Toll-Like Receptor Signaling Genes | Akt1 | Thymoma viral proto-oncogene 1 | NM_009652 |
| | | CASP8 (FLICE) | Caspase 8 | NM_009812 |
| | | Lbp | Lipopolysaccharide binding protein | NM_008489 |
| | | PIK3CA (p110-alpha) | Phosphatidylinositol 3-kinase, catalytic, alpha polypeptide | NM_008839 |
| | | Rac1 | RAS-related C3 botulinum substrate 1 | NM_009007 |
| NOD-Like Receptor (NLR) Signaling | NOD-Like Receptors | NAIP (BIRC1) | NLR family, apoptosis inhibitory protein 1 | NM_008670 |
| | | NLRC4 (IPAF) | NLR family, CARD domain containing 4 | NM_1033367 |
| | | Nlrp1a | NLR family, pyrin domain containing 1A | NM_1004142 |
| | | Nlrp3 | NLR family, pyrin domain containing 3 | NM_145827 |
| | | NOD1 (CARD4) | Nucleotide-binding oligomerization domain containing 1 | NM_172729 |
| | | Nod2 | Nucleotide-binding oligomerization domain containing 2 | NM_145857 |
| | Inflammasomes | Casp1 (ICE) | Caspase 1 | NM_009807 |
| | | Naip1 (BIRC1) | NLR family, apoptosis inhibitory protein 1 | NM_008670 |
| | | NLRC4 (IPAF) | NLR family, CARD domain containing 4 | NM_1033367 |
| | | Nlrp1a | NLR family, pyrin domain containing 1A | NM_1004142 |
| | | Nlrp3 | NLR family, pyrin domain containing 3 | NM_145827 |
| | | PYCARD (TMS1, ASC) | PYD and CARD domain containing | NM_023258 |
| | Regulation of NOD-like Receptor (NLR) Signaling | BIRC3 (c-IAP2) | Baculoviral IAP repeat-containing 3 | NM_007464 |
| | | Card6 | member 6 | NM_1163138 |
| | | Card9 | member 9 | NM_1037747 |
| | | CASP8 (FLICE | Caspase 8 | NM_009812 |
| | | Hsp90aa1 | class A member 1 | NM_010480 |
| | | Mefv | Mediterranean fever | NM_019453 |
| | | Pstpip1 | interacting protein 1 | NM_011193 |

| | | | | |
|---|---|---|---|---|
| | | Ripk1 | threonine kinase 1 | NM_009068 |
| | | Sugt1 | cerevisiae) | NM_026474 |
| | | Tnf | Tumor necrosis factor | NM_013693 |
| | | XIAP (BIRC4) | X-linked inhibitor of apoptosis | NM_009688 |
| Other Bacterial Pattern Recognition Receptors (PRRs) | | Apcs | Serum amyloid P-component | NM_011318 |
| | | Crp | C-reactive protein, pentraxin-related | NM_007768 |
| | | Dmbt1 | Deleted in malignant brain tumors 1 | NM_007769 |
| | | Zbp1 | Z-DNA binding protein 1 | NM_021394 |
| Signaling Downstream of Antibacterial Responses | NFκB Signaling | CHUK (IKKα) | Conserved helix-loop-helix ubiquitous kinase | NM_007700 |
| | | IKBKB (IKKβ) | Inhibitor of kappaB kinase beta | NM_010546 |
| | | Nfkb1 | Nuclear factor of kappa light polypeptide gene enhancer in B-cells 1, p105 | NM_008689 |
| | | NFKBIA (IκBα, MAD3) | Nuclear factor of kappa light polypeptide gene enhancer in B-cells inhibitor, alpha | NM_010907 |
| | | Rela | V-rel reticuloendotheliosis viral oncogene homolog A (avian) | NM_009045 |
| | | TNFRSF1A (TNFR1) | Tumor necrosis factor receptor superfamily, member 1a | NM_011609 |
| | ERK1 / ERK2 Signaling | Jun | Jun oncogene | NM_010591 |
| | | MAP2K1 (MEK1) | Mitogen-activated protein kinase kinase 1 | NM_008928 |
| | | MAPK1 (ERK2) | Mitogen-activated protein kinase 1 | NM_011949 |
| | | MAPK3 (ERK1 | Mitogen-activated protein kinase 3 | NM_011952 |
| | JNK / p38 Signaling | Jun | Jun oncogene | NM_010591 |
| | | Map2k3 | Mitogen-activated protein kinase kinase 3 | NM_008928 |
| | | MAP2K4 (JNKK1) | Mitogen-activated protein kinase kinase 4 | NM_009157 |
| | | MAPK14 (p38ALPHA) | Mitogen-activated protein kinase 14 | NM_011951 |
| | | Mapk8 | Mitogen-activated protein kinase 8 | NM_016700 |
| Apoptosis | | Akt1 | Thymoma viral proto-oncogene 1 | NM_009652 |
| | | BIRC3 (c-IAP2) | Baculoviral IAP repeat-containing 3 | NM_007464 |
| | | Card6 | member 6 | NM_1163138 |
| | | Card9 | member 9 | NM_1037747 |
| | | CASP1 (ICE) | Caspase 1 | NM_009807 |
| | | CASP8 (FLICE) | Caspase 8 | NM_009812 |
| | | Cd14 | CD14 antigen | NM_009841 |
| | | Fadd | Fas (TNFRSF6)-associated via death domain | NM_010175 |
| | | Ifnb1 | Interferon beta 1, fibroblast | NM_010510 |
| | | IKBKB (IKKβ) | Inhibitor of kappaB kinase beta | NM_010546 |
| | | Il12a | Interleukin 12A | NM_008351 |
| | | Il12b | Interleukin 12B | NM_008352 |
| | | Il1b | Interleukin 1 beta | NM_008361 |
| | | Il6 | Interleukin 6 | NM_031168 |
| | | Irak1 | Interleukin-1 receptor-associated kinase 1 | NM_008363 |
| | | Jun | Jun oncogene | NM_010591 |
| | | MAP3K7 (TAK1 | Mitogen-activated protein kinase kinase kinase 7 | NM_172688 |
| | | MAPK1 (ERK2) | Mitogen-activated protein kinase 1 | NM_011949 |
| | | MAPK8 (JNK1) | Mitogen-activated protein kinase 8 | NM_016700 |
| | | Mpo | Myeloperoxidase | NM_010824 |
| | | Nfkb1 | Nuclear factor of kappa light polypeptide gene enhancer in B-cells 1, p105 | NM_008689 |
| | | NFKBIA (IκBα, MAD3) | Nuclear factor of kappa light polypeptide gene enhancer in B-cells inhibitor, alpha | NM_010907 |
| | | PIK3CA (p110-alpha) | Phosphatidylinositol 3-kinase, catalytic, alpha polypeptide | NM_008839 |
| | | PYCARD (TMS1, ASC) | PYD and CARD domain containing | NM_023258 |
| | | Rac1 | RAS-related C3 botulinum substrate 1 | NM_009007 |
| | | Ripk1 | Receptor (TNFRSF)-interacting serine-threonine kinase 1 | NM_009068 |
| | | Ripk2 | Receptor (TNFRSF)-interacting serine-threonine kinase 2 | NM_138952 |
| | | Tnf | Tumor necrosis factor | NM_013693 |
| | | TNFRSF1A (TNFR1) | Tumor necrosis factor receptor superfamily, member 1a | NM_011609 |
| | | Traf6 | Tnf receptor-associated factor 6 | NM_009424 |
| Inflammatory Response | | Akt1 | Thymoma viral proto-oncogene 1 | NM_009652 |
| | | Apcs | Serum amyloid P-component | NM_011318 |

| | | | |
|---|---|---|---|
| | CCL3 (MIP-1A) | Chemokine (C-C motif) ligand 3 | NM_011337 |
| | CCL5 (RANTES) | Chemokine (C-C motif) ligand 5 | NM_013653 |
| | Cd14 | CD14 antigen | NM_009841 |
| | Crp | C-reactive protein, pentraxin-related | NM_007768 |
| | CXCL1 (GRO1, GROa) | Chemokine (C-X-C motif) ligand 1 | NM_001511 |
| | CXCL2 | Chemokine (C-X-C motif) ligand 3 | NM_002089 |
| | Il1b | Interleukin 1 beta | NM_008361 |
| | Il6 | Interleukin 6 | NM_031168 |
| | Lbp | Lipopolysaccharide binding protein | NM_008489 |
| | Ly96 | Lymphocyte antigen 96 | NM_016923 |
| | Lyz2 | Lysozyme 2 | NM_017372 |
| | Mefv | Mediterranean fever | NM_019453 |
| | Myd88 | Myeloid differentiation primary response gene 88 | NM_010851 |
| | Nfkb1 | Nuclear factor of kappa light polypeptide gene enhancer in B-cells 1, p105 | NM_008689 |
| | NLRC4 (IPAF) | NLR family, CARD domain containing 4 | NM_1033367 |
| | Rac1 | RAS-related C3 botulinum substrate 1 | NM_009007 |
| | Nlrp3 | NLR family, pyrin domain containing 3 | NM_145827 |
| | NOD1 (CARD4) | Nucleotide-binding oligomerization domain containing 1 | NM_172729 |
| | Rela | V-rel reticuloendotheliosis viral oncogene homolog A (avian) | NM_009045 |
| | Ripk2 | Receptor (TNFRSF)-interacting serine-threonine kinase 2 | NM_138952 |
| | Slc11a1 | Solute carrier family 11 (proton-coupled divalent metal ion transporters), member 1 | NM_013612 |
| | TICAM2 (TRAM) | Toll-like receptor adaptor molecule 2 | NM_173394 |
| | Tirap | Toll-interleukin 1 receptor (TIR) domain-containing adaptor protein | NM_054096 |
| | Tlr1 | Toll-like receptor 1 | NM_030682 |
| | Tlr2 | Toll-like receptor 2 | NM_011905 |
| | Tlr4 | Toll-like receptor 4 | NM_021297 |
| | Tlr5 | Toll-like receptor 5 | NM_016928 |
| | Tlr6 | Toll-like receptor 6 | NM_011604 |
| | Tlr9 | Toll-like receptor 9 | NM_031178 |
| | Tnf | Tumor necrosis factor | NM_013693 |
| | TNFRSF1A (TNFR1) | Tumor necrosis factor receptor superfamily, member 1a | NM_011609 |
| | Tollip | Toll interacting protein | NM_023764 |
| Cytokines & Chemokines | CCL3 (MIP-1A) | Chemokine (C-C motif) ligand 3 | NM_011337 |
| | CCL5 (RANTES) | Chemokine (C-C motif) ligand 5 | NM_013653 |
| | CXCL1 (GRO1, GROa) | Chemokine (C-X-C motif) ligand 1 | NM_008176 |
| | Ifna9 | Interferon alpha 9 | NM_010507 |
| | Ifnb1 | Interferon beta 1, fibroblast | NM_010510 |
| | Il12a | Interleukin 12A | NM_008351 |
| | Il12b | Interleukin 12B | NM_008352 |
| | Il1b | Interleukin 1 beta | NM_008361 |
| | Il6 | Interleukin 6 | NM_031168 |
| | Il18 | Interleukin 18 | NM_008360 |
| Antimicrobial Peptides | Bpi | Bactericidal permeablility increasing protein | NM_177850 |
| | Camp | Cathelicidin antimicrobial peptide | NM_009921 |
| | Ctsg | Cathepsin G | NM_007800 |
| | Lcn2 | Lipocalin 2 | NM_008491 |
| | Ltf | Lactotransferrin | NM_008522 |
| | Lyz2 | Lysozyme 2 | NM_017372 |
| | Mpo | Myeloperoxidase | NM_010824 |
| | Slpi | Secretory leukocyte peptidase inhibitor | NM_011414 |
| | Prtn3 | Proteinase 3 | NM_011178 |
