# Supplementary material for: Manipulation of Salmonella Typhi Gene Expression Impacts Innate Cell Responses in the Human Intestinal Mucosa
Source: Front Immunol. 2018 Nov 1;9:2543. doi: 10.3389/fimmu.2018.02543 (PMC6221971; doi:10.3389/fimmu.2018.02543)
Supplement: Supplementary file 2 [file Data_Sheet_2.PDF]

Supplemental Table 2. List of the Genes detected by the Tight Junctions RT^2 Profiler PCR Array

Description	Symbol	Description	Refseq
Tight Junctions Molecules	CLDN1	Claudin 1	NM_021101
	CLDN10	Claudin 10	NM_182848
	CLDN11	Claudin 11	NM_005602
	CLDN12	Claudin 12	NM_012129
	CLDN14	Claudin 14	NM_144492
	CLDN15	Claudin 15	NM_014343
	CLDN16	Claudin 16	NM_006580
	CLDN17	Claudin 17	NM_012131
	CLDN18	Claudin 18	NM_016369
	CLDN19	Claudin 19	NM_148960
	CLDN2	Claudin 2	NM_020384
	CLDN3	Claudin 3	NM_001306
	CLDN4	Claudin 4	NM_001305
	CLDN5	Claudin 5	NM_003277
	CLDN6	Claudin 6	NM_021195
	CLDN7	Claudin 7	NM_001307
	CLDN8	Claudin 8	NM_199328
	CLDN9	Claudin 9	NM_020982
	OCLN	Occludin	NM_002538
Cell Adhesion Molecules	ESAM	Endothelial cell adhesion molecule	NM_138961
	ICAM1	Intercellular adhesion molecule 1	NM_000201
	ICAM2	Intercellular adhesion molecule 2	NM_000873
	PECAM1	Platelet/endothelial cell adhesion molecule	NM_000442
Junctional Adhesion Molecules	F11R	F11 receptor	NM_016946
	IGSF5	Immunoglobulin superfamily, member 5	NM_001080444
	JAM2	Junctional adhesion molecule 2	NM_021219
	JAM3	Junctional adhesion molecule 3	NM_032801
Other Cell Receptors	CD99	CD99 molecule	NM_002414
	CRB1	Crumbs homolog 1 (Drosophila)	NM_201253
	CRB3	Crumbs homolog 3 (Drosophila)	NM_139161
Alpha Actinins & Catenins	ACTN1	Actinin, alpha 1	NM_001102
	ACTN2	Actinin, alpha 2	NM_001103
	ACTN3	Actinin, alpha 3	NM_001104
	ACTN4	Actinin, alpha 4	NM_004924
	CTNNA1	Catenin (cadherin-associated protein), alpha 1, 102kDa	NM_001903
	CTNNA2	Catenin (cadherin-associated protein), alpha 2	NM_004389
	CTNNA3	Catenin (cadherin-associated protein), alpha 3	NM_013266
	CTNNB1	Catenin (cadherin-associated protein), beta 1, 88kDa	NM_001904
Junction Associated Proteins	ACTN1	Actinin, alpha 1	NM_001102
	ACTN2	Actinin, alpha 2	NM_001103
	ACTN3	Actinin, alpha 3	NM_001104
	ACTN4	Actinin, alpha 4	NM_004924
	AMOTL1	Angiomotin like 1	NM_130847
	CGN	Cingulin	NM_020770
	YBX3	Cold shock domain protein A	NM_003651
	CTTN	Cortactin	NM_005231
	EPB41	Erythrocyte membrane protein band 4.1 (elliptocytosis 1, RH-linked)	NM_004437
	HCLS1	Hematopoietic cell-specific Lyn substrate 1	NM_005335
	INADL	InaD-like (Drosophila)	NM_176877
	MAGI1	Membrane associated guanylate kinase, WW and PDZ domain containing 1	NM_004742
	MAGI2	Membrane associated guanylate kinase, WW and PDZ domain containing 2	NM_012301

	MLLT4	Myeloid/lymphoid or mixed-lineage leukemia (trithorax homolog, Drosophila); translocated to, 4	NM_001040000
	MPDZ	Multiple PDZ domain protein	NM_003829
	PARD3	Par-3 partitioning defective 3 homolog (C. elegans)	NM_019619
	SYMPK	Symplekin	NM_004819
	TIAM1	T-cell lymphoma invasion and metastasis 1	NM_003253
	TJAP1	Tight junction associated protein 1 (peripheral)	NM_080604
	TJP1	Tight junction protein 1 (zona occludens 1)	NM_175610
	TJP2	Tight junction protein 2 (zona occludens 2)	NM_004817
	TJP3	Tight junction protein 3 (zona occludens 3)	NM_014428
	VAPA	VAMP (vesicle-associated membrane protein)-associated protein A, 33kDa	NM_194434
	ZAK	Sterile alpha motif and leucine zipper containing kinase AZK	NM_016653
Cytoskeleton Regulators	AMOTL1	Angiomotin like 1	NM_130847
	ASH1L	Ash1 (absent, small, or homeotic)-like (Drosophila)	NM_018489
	YBX3	Cold shock domain protein A	NM_003651
	CTTN	Cortactin	NM_005231
	LLGL1	Lethal giant larvae homolog 1 (Drosophila)	NM_004140
	LLGL2	Lethal giant larvae homolog 2 (Drosophila)	NM_004524
	PARD3	Par-3 partitioning defective 3 homolog (C. elegans)	NM_019619
	PARD6A	Par-6 partitioning defective 6 homolog alpha (C. elegans)	NM_016948
	PARD6B	Par-6 partitioning defective 6 homolog beta (C. elegans)	NM_032521
	SMURF1	SMAD specific E3 ubiquitin protein ligase 1	NM_020429
	SPTA1	Spectrin, alpha, erythrocytic 1 (elliptocytosis 2)	NM_003126
	SPTAN1	Spectrin, alpha, non-erythrocytic 1 (alpha-fodrin)	NM_003127
	SPTB	Spectrin, beta, erythrocytic	NM_000347
	TIAM1	T-cell lymphoma invasion and metastasis 1	NM_003253
G-Protein Signaling	ARHGEF2	Rho/rac guanine nucleotide exchange factor (GEF) 2	NM_004723
	CDC42	Cell division cycle 42 (GTP binding protein, 25kDa)	NM_001791
	CDK4	Cyclin-dependent kinase 4	NM_000075
	GNAI1	Guanine nucleotide binding protein (G protein), alpha inhibiting activity polypeptide 1	NM_002069
	RAC1	Ras-related C3 botulinum toxin substrate 1 (rho family, small GTP binding protein Rac1)	NM_006908
	RHOA	Ras homolog gene family, member A	NM_001664
	SMURF1	SMAD specific E3 ubiquitin protein ligase 1	NM_020429
	TIAM1	T-cell lymphoma invasion and metastasis 1	NM_003253
Protein Kinase Signaling	CASK	Calcium/calmodulin-dependent serine protein kinase (MAGUK family)	NM_003688
	CSNK2A1	Casein kinase 2, alpha 1 polypeptide	NM_001895
	CSNK2A2	Casein kinase 2, alpha prime polypeptide	NM_001896
	CSNK2B	Casein kinase 2, beta polypeptide	NM_001320
	ILK	Integrin-linked kinase	NM_004517
	MAGI1	Membrane associated guanylate kinase, WW and PDZ domain containing 1	NM_004742
	MAGI2	Membrane associated guanylate kinase, WW and PDZ domain containing 2	NM_012301
	MARK2	MAP/microtubule affinity-regulating kinase 2	NM_004954
	MPP5	Membrane protein, palmitoylated 5 (MAGUK p55 subfamily member 5)	NM_022474
	MPP6	Membrane protein, palmitoylated 6 (MAGUK p55 subfamily member 6)	NM_016447
	PRKCI	Protein kinase C, iota	NM_002740
	PRKCZ	Protein kinase C, zeta	NM_002744
	PTEN	Phosphatase and tensin homolog	NM_000314
